# Integration of Biosensors and Drug Delivery Technologies for Early Detection and Chronic Management of Illness

**DOI:** 10.3390/s130607680

**Published:** 2013-06-14

**Authors:** Mpho Ngoepe, Yahya E. Choonara, Charu Tyagi, Lomas Kumar Tomar, Lisa C. du Toit, Pradeep Kumar, Valence M. K. Ndesendo, Viness Pillay

**Affiliations:** 1 Department of Pharmacy and Pharmacology, Faculty of Health Sciences, University of the Witwatersrand, 7 York Road, Parktown, 2193, Johannesburg, South Africa; E-Mails: mpho.ngoepe@students.wits.ac.za (M.N.); yahya.choonara@wits.ac.za (Y.E.C.); lisa.dutoit@wits.ac.za (L.C.D.); pradeep.kumar@wits.ac.za (P.K.); charu.tyagi@wits.ac.za (C.T.) lomaskumar.tomar@wits.ac.za (L.K.T.); 2 School of Pharmacy and Pharmaceutical Sciences, St. John's University of Tanzania, Dodoma, Tanzania; E-Mail: vndesendo@sjut.ac.tz

**Keywords:** biosensor, BioMEMS, biomarkers, closed loop system, illness management, implantable systems

## Abstract

Recent advances in biosensor design and sensing efficacy need to be amalgamated with research in responsive drug delivery systems for building superior health or illness regimes and ensuring good patient compliance. A variety of illnesses require continuous monitoring in order to have efficient illness intervention. Physicochemical changes in the body can signify the occurrence of an illness before it manifests. Even with the usage of sensors that allow diagnosis and prognosis of the illness, medical intervention still has its downfalls. Late detection of illness can reduce the efficacy of therapeutics. Furthermore, the conventional modes of treatment can cause side-effects such as tissue damage (chemotherapy and rhabdomyolysis) and induce other forms of illness (hepatotoxicity). The use of drug delivery systems enables the lowering of side-effects with subsequent improvement in patient compliance. Chronic illnesses require continuous monitoring and medical intervention for efficient treatment to be achieved. Therefore, designing a responsive system that will reciprocate to the physicochemical changes may offer superior therapeutic activity. In this respect, integration of biosensors and drug delivery is a proficient approach and requires designing an implantable system that has a closed loop system. This offers regulation of the changes by means of releasing a therapeutic agent whenever illness biomarkers prevail. Proper selection of biomarkers is vital as this is key for diagnosis and a stimulation factor for responsive drug delivery. By detecting an illness before it manifests by means of biomarkers levels, therapeutic dosing would relate to the severity of such changes. In this review various biosensors and drug delivery systems are discussed in order to assess the challenges and future perspectives of integrating biosensors and drug delivery systems for detection and management of chronic illness.

## 1. Introduction

Management of chronic illnesses such as diabetes and cardiovascular disorders require maintenance of glucose and cholesterol levels. For better health management, biological sensors or biosensors have been used in diagnostics. Biosensors are analytical devices that utilize biological recognition elements such as antibodies and receptors for the detection of disease biomarkers, followed by the quantification of biomarkers by means of transducers [2,3]. Once the state and level of disease/illness is assessed, it is then followed by prognosis and finally illness management with intervention of suitable therapeutics. Drug delivery systems offer illness management by means of utilizing sustainable responsive and targeted drug delivery vehicles. These procedures followed by illness management are expensive as they require highly skilled personnel and expensive equipment. In many cases, medical intervention efficiency is reduced when the most high risk (susceptible) patients are not diagnosed at an early stage such as in the case of cancer and cardiovascular diseases. Biomarkers which are measurable and quantifiable biological parameters such as macromolecule concentration, volatile compounds and genetic variation (single nucleotide polymorphism), found in the presence of biological material, serve as indicators for health and physiology-related assessments [4]. Selection of biomarkers is therefore the key to illness management before they manifests. In this review various biomarkers, biosensors and drug delivery systems will be discussed in order to improve diagnostics and therapeutic intervention by integrating biosensors with drug delivery system. This can help improve chronic illness caused by glucose and cholesterol.

The diagnosis of profound disease like cancer generally requires a biopsy to be performed. This is however an invasive procedure where prognosis is limited. Accurate analysis requires technologies such as micro arrays in order to trace susceptibility and level of severity. The analysis of proteomics and genomics require sophisticated instruments and highly trained personnel for data analysis in relation to the high number of people diagnosed with cancer. As an alternative, physiochemical changes that occur during illness can be analyzed making use of noninvasive procedures. Lung cancer is one of the illnesses that can be diagnosed by means of analyzing exhaled volatile organic compounds [5,6]. Volatile organic compounds such as hexane, methylpentane and benzene derivatives such as *o*-toluidine and aniline have been used as lung cancer biomarkers [7,8]. This technology will replace the use of X-rays which does not show illness manifestation until a tumor has formed.

**Figure 1.**
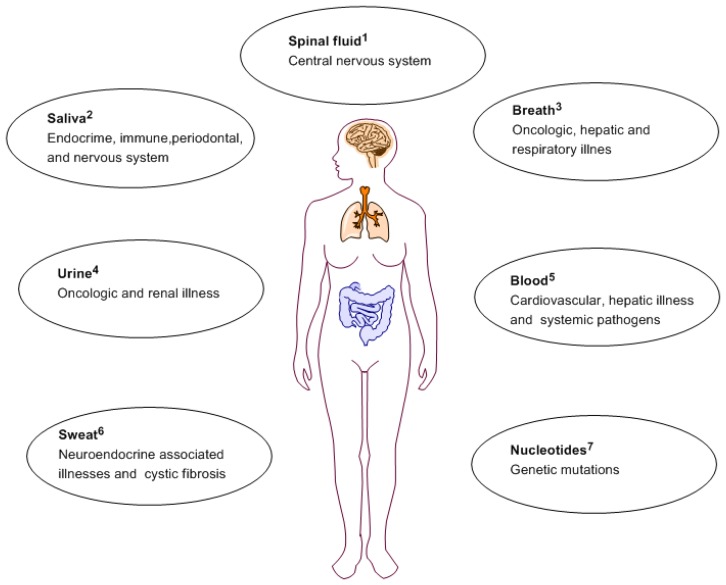
Routes of obtaining biomarkers for a variety of illnesses. 1. Spinal fluid [11]; 2. Saliva [12]; 3. Breath [13]; 4. Urine [14]; 5. Blood [15]; 6. Sweat [16]; 7.Nucleotides [17].

For cardiovascular disease, biomarkers can be useful tools for better identification of susceptible individuals, early disease diagnosis and offer an inter-individual prognosis and illness management [9]. The related susceptibility includes disorders of the blood vasculature and the heart, and even stroke due to late diagnosis [10]. Cardiovascular disease diagnosis can vary amongst individuals based on age, sex and body mass index. Portable biosensors devices able to detect specific biomarkers can be used by patients to monitor their health on daily basis. Figure 1 depicts routes of obtaining biomarkers for a variety of illnesses.

Recent advances in oral biology have associated changes in salivary contents with local and systemic illness. Diabetes, cystic fibrosis, periodontal illness and several other illnesses have been demonstrated to express biochemical markers in the saliva and exhaled breath [18]. Saliva offers a great advantage over other sampling methods as it is readily accessible via a totally non-invasive method [19,20]. However due to the low concentrations of analytes present in saliva, this requires very sensitive detection systems. Other oral studies have shown that presence of certain volatile organic compounds (VOCs) in exhaled human breath can act as biological signatures of specific pathophysiological conditions [21–24]. After diagnosis, medical intervention is required for illness management. This is where drug delivery plays an important role since proper mode of delivery contributes substantially to the efficiency of illness management.

Advances in drug delivery systems and technologies aim at overcoming limitations of conventional drug delivery using traditional dosage forms by achieving enhanced bioavailability and therapeutic index, reduced side effects, and improved patient acceptance or compliance [25]. The purpose of modern drug delivery systems is to improve the pharmacokinetics and pharmacodynamics which often play very important roles in therapeutic efficacy and overall functioning of the body systems. Pharmacokinetics deals with drug delivery inside the body, which involves absorption, distribution, metabolism and elimination, while pharmacodynamics deals with the physiological effects of drugs on the body and the mechanisms of drug action. The relationship between drug concentration and effects require efficient formulation to meet optimum illness management. The use of responsive polymers, microtubules and nanoparticles has allowed targeting sites of illness and controlling the drug release profile. Factors exerting crucial role in drug delivery system design are biocompatibility, controlled drug release and degradation.

Integration of diagnosis and therapeutics into a single system can improve illness management. Combination of biosensors and drug delivery system vehicles does not only allow self-regulated therapeutics but is a protective means against biohazard agents as well [26]. Detection of biohazards levels, chemical and biochemical substances require selection of a marker which can be used as direct or indirect indicator. As in environmental applications pollution can be determined by detecting the level of elevated foreign compounds and chemical by-products, the same mode of detection is applied for illness management. Biochemical imbalances, such as those of glucose and cholesterol levels, are indicative of different illness; hypercholesterolemia and hyperglycemia signify elevated levels of cholesterol and glucose, whereas hypercholesterolemia and hypoglycemia indicate their low levels.

The identification of a chemical, biochemical or pathogen organism is generally been done employing common technologies of ELISA, PCR, flow cytometry and spectroscopy. These are time consuming, require specialized training, and involve complicated processing steps to culture or extract the analyte from samples. In relation to drug delivery, conventional modes of delivery such as oral, rectal, transdermal, subcutaneous, or sublingual administration have shown lower bioavailability (depends on the chemical nature of the administered compound such as hydrophilic or hydrophobic), whereas intravenous and intramuscular routes of administration have shown to reduce patient compliance [27]. Implantable and portable biosensors for drug delivery offer self-monitoring and increased patients' compliance [28]. Integrated biosensors and drug delivery devices can offer a continuous diagnosis, prognosis and efficient therapeutic management.

## 2. The Role and Rationale of Biosensors in Illness Management

Biosensors can be used to monitor physiochemical changes in the body with high sensitivity and specificity. This offers a powerful opportunity in early diagnosis and treatment of illness. Early detection and diagnosis can greatly reduce the cost of patient medical care, associated with advanced stages of many illnesses and far better can prevent an illness before it manifests. From a diagnostics view point, accuracy of the diagnosis is vital in terms of the kind of therapeutic to be used. The major concern in diagnosis is patient compliance where invasive samples (blood and tissue) are routinely taken to analyze the severity of the illness. In terms of prognosis, there are limitations since it is not accurate to estimate the likely outcome of the illness in an individual. Both these affect illness management since dosage and period of treatment affect the level of illness, patient compliance and medical costs. Thus, for chronic illness, continuous medical intervention is required to allow changing of the dosage and treatment period. This may evidently be observed in diabetes management where treating hyperglycemia can lead to hypoglycemia.

Imbalances of glucose and cholesterol are a major concern since they are the major cause of fatal illnesses. Glucose imbalance leads to diabetes. This even increases the risk of heart diseases, kidney failure, and/or blindness [29]. Both, high and low levels of glucose can result in disability or death. From the diagnosis and management point of view, diabetes mellitus requires a continuous monitoring of blood glucose levels. In 2012 glucose biosensors accounted for approximately 85% of the world market for biosensors [30]. Millions of diabetics test their blood glucose levels daily, thus making glucose the most commonly tested analyte. The first biosensor created and reported was for this analyte glucose in 1962 by Clark and Lyons [31], where glucose oxidase enzyme was entrapped on an oxygen electrode over a semi-permeable dialysis membrane. Glucose levels were indirectly measured by detecting the amount of oxygen consumed by the enzyme. In 1973, Guilbault and Lubrano [32] designed an amperometric (anodic) sensor to monitor the hydrogen peroxide, a glucose degradation byproduct.

Prior to any major impacts, an illness can cause serious problems to the patient such as neuropathy or retinopathy in terms of diabetes, since there are a number of physiochemical changes which occur. Diabetic retinopathy which occurs due to low sugar levels in the eyes can lead to blindness as the new capillaries that deliver blood to the eye are fragile [33]. For cholesterol, there are a variety of physiochemical changes that occur before signifying future damage. Blood clogging precedes the occurrence of ultimate stroke causing the interference of blood flow near the nervous system. Prognosis of any illness plays a major role in illness management. However, through the process of diagnosis, chronic illnesses will require continuous monitoring for efficient management. The costs and patients compliance are highly affected by these processes. It is observed that self-monitoring of sugar levels has benefited patients in terms of costs and disease management. Design of self-monitoring devices for glucose levels such as SensoCard Plus (BBI Healthcare) and AccuCheck Compact (Roche) has aided patients to monitor their glucose concentrations in order to delay or even prevent the progression of microvascular and macrovascular complications [34]. The mode of self-monitoring indeed has some substantial role since it affects compliance in terms of monitoring analyte concentration daily without causing discomfort to the patient. For better illness management, non-invasive/continuous sampling is required for optimum medical intervention. There are different kinds of glucose sensors which can be divided into two groups; enzymatic (finger-prick glucometer and urine dipstick), and continuous (non-invasive, minimally invasive and invasive). For continuous invasive sensors, these can be intravenous, implantable, microdialysis (glucose oxidase electrochemical sensor) and subcutaneous sensors (enzyme electrodes-redox reaction analysis), while for minimally invasive, micropore or microneedle (collection of interstitial fluid for enzyme based electrode sensor) can be used [35].

For cholesterol management, it has been shown that cumulative treatment discontinuations among long-term regimens of all types of drugs is about 50% of patients during first year and 85% of patients in the second year of treatment [36]. Similar to diabetes, cholesterol is detected by means of using immobilized enzymes (CholesTrak^®^, *A*ccuTech, LLC). The enzyme cholesterol oxidase breaks down cholesterol into hydrogen peroxide and cholest-4-en-3-one in the presence of oxygen [37]. The level of cholesterol is then measured by an amperometric sensor that can detect hydrogen peroxide through redox mediator [38]. The use of enzymes for detection of any analyte faces disadvantages such as short lifetime and lower sensitivity. This can be avoided by using two or more enzymes. In the case of cholesterol, cholesterol oxidase and cholesterol esterase can be used in combination [39]. Future cholesterol monitoring devices may include non-invasive mode of cholesterol level detection as in quantifying the levels of isoprene in human breath [40].

For non-invasive sensors the mode of detection can be either optical or via transdermal analysis. For transdermal analysis, impedance spectroscopy (dielectric properties of a tissue), skin suction blister technique (vacuum application on the skin to obtain fluid for analysis), reverse iontophoresis (low electric current application) and sonophoresis (use ultrasound on the skin) may be used [41,42]. For optical analysis the following methods may be employed; kromoscopy (electromagnetic radiation), photoacoustic spectroscopy (increased ultrasound pulse generated during absorption of light when there is high glucose levels), optical coherence tomography (tomographic imaging), scattering (relative refractive indices of a particle), occlusion spectroscopy (produce high systolic pressure to occlusion of arterial flow), polarimetry (substances which can rotate the plane of polarized light), thermal infrared (glucose concentration correlates to temperature variation and MIR light scattering on the skin), fluorescence (light emission from molecules in different states), MIR spectroscopy (wavelength variations due to stretching and bending of molecules), NIR spectroscopy (absorption based on molecular structure) and Raman spectroscopy (rotational or vibrational energy states within a molecule) [43].

## 3. Biosensors

**Figure 2.**
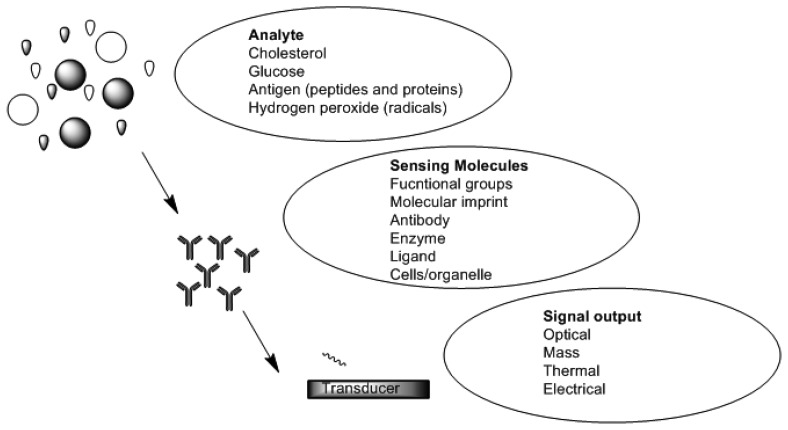
Schematic depicting functional principles of a biosensor.

Chemical sensors and biosensors are of interest within the field of modern analytical chemistry and pharmaceutics. There are a number of published research works which show the diversity of approaches and techniques applied. This is due to new demands and opportunities that are appearing particularly in clinical diagnostics, environmental analysis, food analysis and production monitoring [44–46]. A sensor is a device which functions by producing a signal which is proportional to the concentration of a specific (bio) chemical or a set of (bio) chemicals in the presence of a number of interfering species [47]. This is accomplished by means of using biological recognition elements such as enzymes, antibodies, receptors, tissues and microorganisms as sensitive materials because of their excellent selective functionality for target substances. Figure 2 is a schematic depicting functional principles of a biosensor. Sensors can be divided into various groups based on the mode of function in terms of sensing region and transduction.

### 3.1. Immunosensor and other Affinity Biosensors

**Figure 3.**
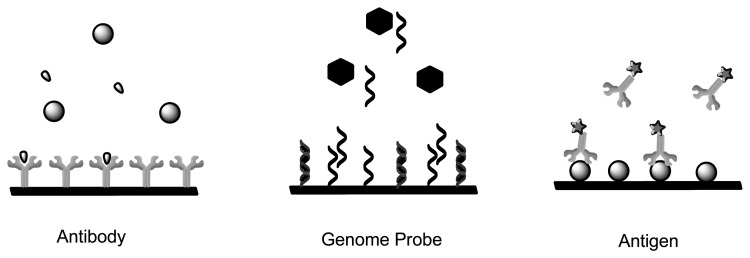
A schematic depicting antibodies and antigens as immunosensor prototypes and genome probe as genosensor prototype (Adapted from [53]).

Immunosensors and Affinity Biosensors constitute immobilized biological recognition elements such as antibodies, antigen, receptor protein and short oligonucleotide sequences for detection of biomarkers [48–50]. Once the analyte binds to the sensing element, the signal is converted by the transducer into a measurable unit. The mode of quantification can be achieved by measuring the specific activity of a label, such as its radioactivity, enzyme activity, fluorescence, chemi-luminescence or bioluminescence [51,52]. Immunosensors use antibodies or antibody fragments as biological recognition fragments which generate a signal during physical changes that occur due to immune complex formation (Figure 3). Single-stranded oligonucleotide sequences known as aptamers are also considered immunosensors as they mimic antibodies properties by being folded into order to form structures that allow binding to target analytes [49]. Contrary to aptamers, genome sensors use probes (nucleic acid fragments) which specifically recognize and bind to a complementary/target nucleic acid strand. The recognition is dependent upon the formation of stable hydrogen bonds between the two nucleic acid strands due to nucleotides hybridization. Hydrophobic, ionic and hydrogen bonds play a role in both genome sensors and immunosensors [53]. Both these applications can be used to detect degree of viral infection and forms of cancer (microarray-mRNA) whereby the immunosensor would detect structural components of the virus, whereas genome sensors would detect the genomic fingerprint [54].

Depending on the method of signal transduction, sensors can further be divided into four basic groups: optical (bioreporter), mass (cantilever), electrochemical (amperometric), and thermal sensors [55]. Electrochemical biosensors can thus be classified as either being biocatalytic (enzyme) or affinity (antibody) devices [56].

Table 1 is a classification of the various groups of biosensors (based on transduction signal) their mode of detection and application. Electrochemical and mass sensitive biosensor are the mostly used for detection and diagnosis of chronic disease as analytes can be obtained in a non-invasive manner.

#### 3.1.1. Amperometric Immunosensors

**Figure 4.**
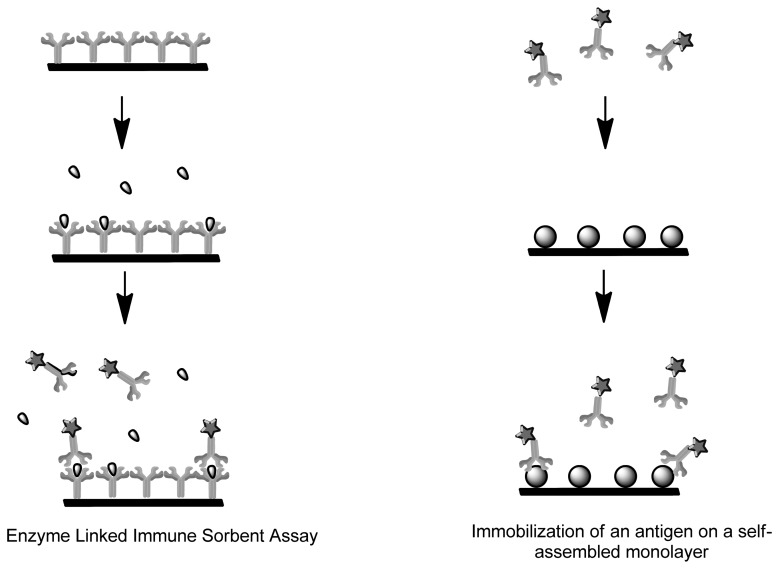
Amperometric immunosensor based on a new electrochemical detection scheme (adapted from [75]).

Enzyme-linked immunosorbent assay (ELISA) represents an amperometric immunosensor where the enzymes undergo redox reactions to generate an electrochemically active product (Figure 4). Current amperometric immunosensors use antibodies or antigens due to their high sensitivity. They can be immobilized onto polymer membrane, Langmuir-Blodgett film, sol-gel and self-assemble monolayers. Unlike enzymes, the antibodies and antigens lack electrochemical activity, therefore for functionality in biosensing they have to be labeled or use a probe molecule such as ferricyanide in the solution [73]. Carcinoembryonic antigens (CEA) are extensively studied biomarker for tumor. An amperometric biosensor was designed for detection of the antigen by means of immobilization of anti-CEA monoclonal antibody on a self-assembled monolayer [74]. There are two different kinds of immunoassays, the homogeneous immunoassay which involves a mixture of antibodies, antigens and labeled antigens. The antigens can be distinguished by a change of activity of the marker when coupled during competitive binding. Heterogeneous immunoassays have antibody or antigen immobilized on a solid support where the immune-complex forms when a solution containing the other immuno agent is added [75]. The disadvantage is that labeling is a complicated and time-consuming process that often leads to physiologically irrelevant binding information and the denaturation of the modified proteins [76].

#### 3.1.2. Label Free Immunosensors

**Figure 5.**
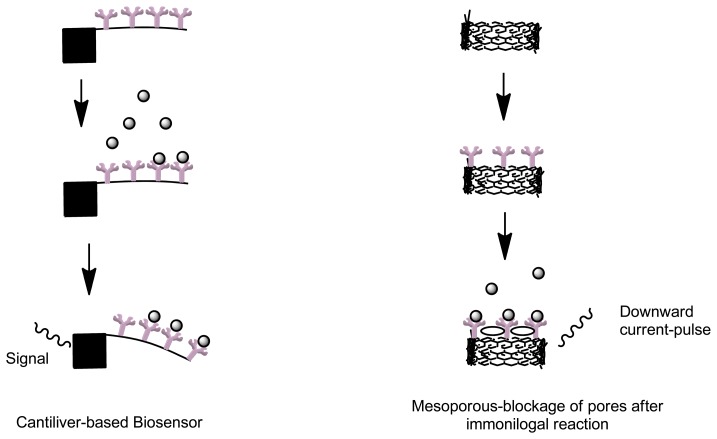
A schematic depicting the prototype label free immunosensor (adapted from [80]).

Cantilevers are an example of label free biosensors which offer a simple, rapid, reliable, minimal cost and low limit of analyte detection (Figure 5). Due to its label free detection principle and small size, this type of biosensor has applicable advantages in diagnostic applications, disease monitoring and research in genomics and proteomics [77]. A cantilever biosensor functions by means of transduction of the molecular interaction between analyte and capturing molecule, immobilized as a layer on one surface of a cantilever. Biomolecular interactions taking place on a solid-state interface leads to an increase in mass [78]. This process results in bending of the cantilever. The capturing molecules are immobilized onto the cantilever by means of direct absorption or by means of covalent attachment to the surface modified with functional groups [79]. Other label-free immunosensors include optical label free detectors such as venerable surface plasmon resonance sensors that can obtain quantitative data on intermolecular binding [60]. Label-free voltammetric immunosensors use electro-active residues in the antibody structure to give specific current response during immune complex formation [73]. Carbon nanotubes and self-assembled monolayer (SAM) represent some of label free biosensors [73]. Apart from label-free measurements that utilize detection of refractive index with surface plasmon resonance, mass change with quartz crystal microbalance and change in conductivity, viscosity and mass with surface acoustic wave; a novel method that utilizes the use changes in ion channels current can be used. By means using a mesoporous polymer, when an analyte of interest enters into the polymer nanopore, this will transiently block the ion current, resulting in a downward current-pulse. Through this mechanism, analytes detection can be achieved by monitoring the blockage of nanopores before and after an immunological reaction as the current-pulse frequency is proportional to the concentration of the analyte [80].

### 3.2. Bioreporter Type Biosensors

**Figure 6.**
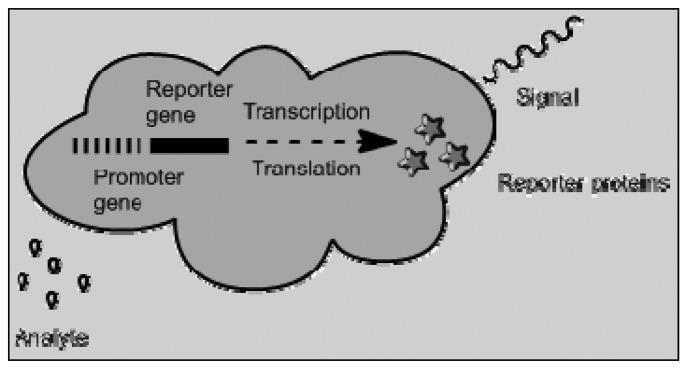
Mechanism of bioreporters (adapted from [81]).

Bioreporters is a fusion of genome biosensors and cell based sensors. Genetically modified microbial can be used to produce a measurable signal in response to a specific chemical or physical agent in their environment [81]. Cell-based biosensors have been used in various fields such as biomedicine, environmental monitoring and pharmaceutical screening. They offer high sensitivity, excellent selectivity and rapid response. In pharmaceutics, these biosensors are useful in analyzing the effect of pharmaceutical compounds on a given physiological system. Enzyme-based biosensor can also be classified with genome and cell-based sensors and can be used to convert the analyte into a quantifiable substance exhibiting fluorescence or conductivity. Figure 6 shows a schematic depicting the mechanism by which bioreporters are able to produce a measurable signal. The reporter proteins range from green fluorescent protein, aequorin, firefly luciferase, and/or bacterial luciferase [82].

### 3.3. Enzyme-Based Biosensors (Electrochemical Biosensors)

Enzymatic activities tend to either produce or consume protons and/or electroactive species [83]. The use an electrode as the transducer can be utilized to quantify the amount analytes during enzymatic reaction. Biosensors constituting enzymes usually employ a class of enzymes known as oxidoreductases, whilst in some case oxidases and dehydrogenases can also be used [83]. When direct transfer of electron between the electrode and enzyme redox center cannot be accomplished, this requires the use of a mediator (must be non-toxic, independent of the pH, stable in both the oxidized and reduced forms) such as ferrocene which can aid in promoting the relay of electron transfer to an electrode [84]. In another study it was found that dopamine and daunomycin can improve the relay [85]. Other mediators involve the use of organometal compounds [38]. In enzyme-based biosensor, the presence of oxygen affects the activity of the mediator. Therefore the use of mediators improves biosensor performance by eliminating the oxygen dependence and improves the ability to control the concentration of the oxidizing agent in the biosensor [56,86]. The use of enzyme electrodes as biosensors will continue to increase because they are simple and inexpensive to manufacture, and they provide rapid analysis with the possibilities of being easily regenerated and reusable [87,88].

#### 3.3.1. Glucose Biosensors

**Figure 7.**
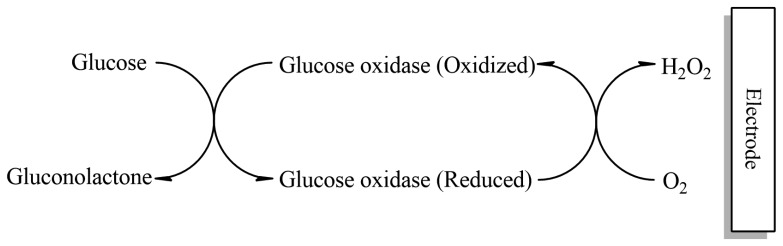
A schematic depicting the basic mechanism of glucose sensor [43]. Commercial glucose biosensors: Ultimate EZ Smart Plus test strips (EZ Smart) and Contour blood glucose test strips (Bayer Healthcare LCC).

Electrochemical oxidation of glucose has been extensively studied for applications in glucose–oxygen fuel cells and in glucose sensors [89]. Glucose oxidase biosensors (GOx) are used to convert glucose into hydrogen peroxide, which in turn can electrochemically be detected with the electrochemical/amperometric transducer [90,91]. Figure 7 is a schematic depicting the basic mechanism of a glucose sensor. In this sensor, glucose is oxidized into gluconolactone at the membrane, a process that involves the consumption of oxygen (O_2_). Hydrogen peroxide (H_2_O_2_) is produced at the same time. Both O_2_ and H_2_O_2_ can be measured by the electrode. The electrocatalytic oxidation of glucose in alkaline medium was investigated using copper, nickel, iron, platinum and gold electrodes. Gold is more favorable metal for the oxidation of sugars, because its oxidation potential in neutral and alkaline medium is more negative compared to the other metals [92].

#### 3.3.2. Cholesterol Biosensor

**Figure 8.**
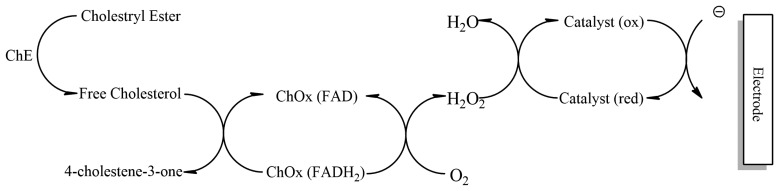
A schematic showing an immobilized enzyme biosensor (adapted from [96]). Commercial cholesterol biosensors: CardioChek Cholesterol meter and Cholesterol Biometer cholesterol (Polymer Technology Systems, Inc.).

Cholesterol oxidase contains flavin adenine dinucleotide (FAD) as the active redox centre. During enzymatic reaction, oxygen acts as a physiological mediator on the electrode surface which undergoes electrochemical oxidation and leads to formation of cholest-4-enone and hydrogen peroxide [93]. The increase in H_2_O_2_ or reduction in O_2_ can be used to determine the amount of cholesterol. This however has a disadvantage as the variation in oxygen tension of the sample leads to fluctuations in electrode response while reoxidation of hydrogen peroxide leads to increased interference from metabolites such as ascorbate and uric acid [94]. To overcome this disadvantage a combination of two or more enzymes is used which offer more selectivity for the analyte (primary enzyme cholesterol oxidase acts on cholesterol, generated hydrogen peroxide caught by a secondary enzyme peroxidase or hemoglobin) of interest and reduce chances of interference [95]. A disposable biosensor has been developed that can determine total cholesterol (Figure 8). The total cholesterol is determined by disposable strips immobilized with Fe_3_O_4_, cholesterol oxidase (ChOx) and cholesterol esterase (ChE) [96]. The enzyme combination allows the detection of both esterified and free cholesterol.

### 3.4. Imaging-Based Biosensors

Imaging can play an important role in diagnosis and treatment. When dealing with cancer, there are number of problems which may occur when using conventional methods such as surgery, chemotherapy and radiation therapy. There are limitations and drawbacks to these modes of treatments mainly due to limited early diagnosis, nonspecific drug distribution, systemic toxicity and unpredictable pharmacodynamics and pharmacokinetics [97]. During surgery, imaging would allow tracing cancer cells that are still localized in the body, and this can even be useful during biopsy operations. For chemotherapy, carrier functionality would be beneficial as it offers target specificity and controlled drug release. Before the radiation therapies it is important to identify the target region.

**Figure 9.**
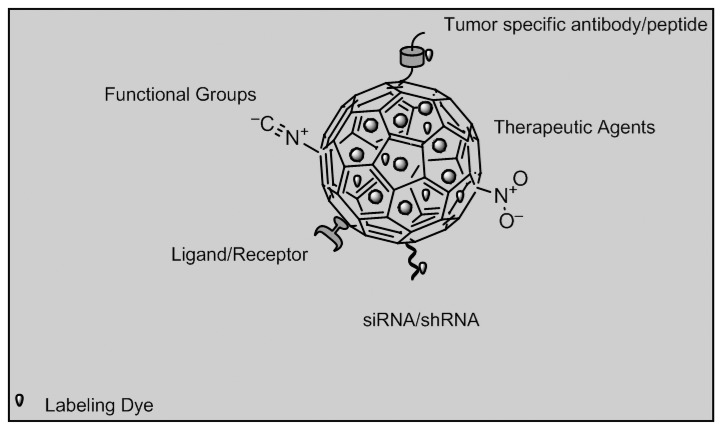
Functionalized nanoparticles used in imaging biosensors (adapted from [99]).

Nanoparticle imaging would prevent radiation damage to other tissues around the target area, thus offering better therapeutic targeting. Targeting and controlled drug release will improve illness management by interfering with illness progression, while biosensor will affect illness diagnosis and prognosis [98]. Organic dye dope nanoparticles made of silica, poly (D,L-lactic-co-glycolic acid) or PLGA and doped with dyes such as IRG-023 Cy5, fluorescein isothiocyanate (FITC) and rhodamine B isothiocyanate (RITC) can be used. Quantum dots are semiconductor crystals composed of elements from groups II to VI, III to IV or IV to VI from the periodic table while up-conversion nanoparticles are synthesized from LaF_3_, YF_3_, Y_2_O_3_, LaPO_4_, NaYF_4_ co-doped with trivalent rare earth ions such as Yb^3+^, Er^3+^ and Tm^3+^[97]. Other groups of imaging biosensors involve multifunctional nanoparticles which can be divided into metallic nanoparticles such as paramagnetic nanoparticles used in cancer therapy, liposome and dendrimers used in cancer and HIV therapy [99]. Figure 9 depicts nanoparticles functionalized with different strategies that can be possibly be used in imaging biosensors of cancer biomarkers such as estrogen, progestogen receptors and occurrence of lethal phenotypes.

## 4. Drug Delivery Systems

Drug delivery system platform is a rapidly expanding market for pharmaceutical and biomedical engineering. In terms of pharmaceuticals, the need for drug carriers that will offer targeted drug delivery is of vital importance. This is of great value as it reduces the side effect profile by allowing usage of low dosage drugs, site specific activity and increased bioavailability. Non-targeted systemic drug administration leads to the bio-distribution of pharmaceuticals across the entire body [100]. This distribution causes toxicity effects on non-target tissues and wastage of pharmaceutical compounds since they are used by non-target tissues. For biomedical engineering, design of devices that will offer better diagnosis and therapeutics is required to ensure better illness management. Biomedical engineering will aid in targeted drug delivery, selective targeting of imaging contrast agents, delivery of nucleic acid and genetic therapies, and prediction of pharmacokinetics and pharmacodynamics patterns of the drug [101]).

**Figure 10.**
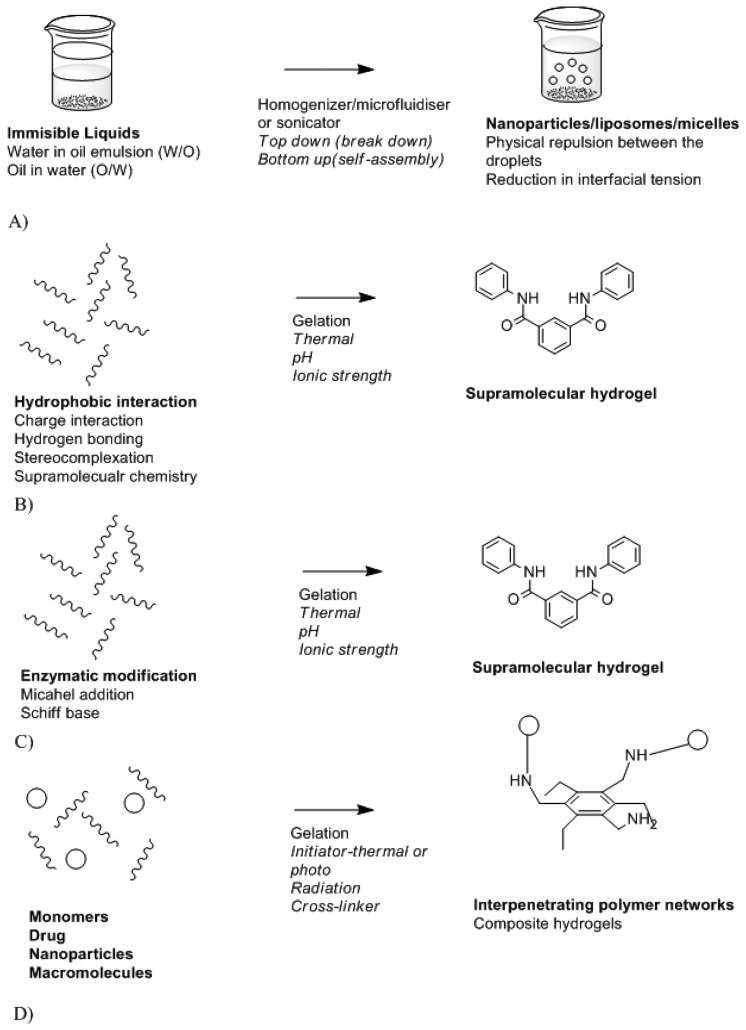
Different modes of drug delivery system synthesis. A. Nanoparticles/macroparticle/liposome formation [106]; B. Physically cross-linked hydrogels [107]; C. Chemically cross-linked hydrogels [108]; D. Polymerization/grafting/molecular imprint [109].

Biomaterials are needed to design a stable and biocompatible drug delivery system. These can vary from natural polymers, metals compound, modified and synthetic polymers. Biocompatibility and biodegradation of these play a vital role in the toxicity effect of the system and its mode of action. A beneficial drug delivery system must have an effect on drug absorption, distribution, and metabolism levels [102]. This can be achieved by controlling drug delivery system. Controlled drug delivery systems function by means of controlling where and when the therapeutic agent will be released. The major features of controlled drug delivery system include the rate of drug release and mode of activation. Drug release may be rapid or may occur over a prolonged period of time depending on the required action and the location of the device in the body. Figure 10 is a schematic depicting the different modes of drug delivery system synthesis while Table 2 provides the classification of drug delivery system platforms.

The mode of release and the rate is related to the biomaterial constituting the major part of the system. Depending on the location where the system is directed to release the drug, the biomaterial that make up the system play a role in terms of reacting with the physiochemical compounds to protect the therapeutics, sense the activator and also allow binding to the target site for localized drug release. Targeted drug delivery can be done by means of using natural organic compounds. These natural compounds interact with surface of the synthetic/modified polymers and peptides. The use of sugar molecules which can be mucoadhesive allows targeting of the intestine. These will be stimulated by temperature (e.g., poly (*N*-isopropylacrylamide)) and pH level (polyacrylic acid and chitosan) for drug release. There are different kinds of polymers that can be used for this purpose; anionic (polyacrylic acid), cationic (chitosan), non-ionic (polyethylene glycols) and thiolated polymers (cysteine conjugates) [103].

Depending on the mode of action required for the drug delivery system, these biomaterials can be modeled into different forms such as spheres for carrying therapeutics and film/hydrogels layers for physiochemical response. For therapeutic implication, nanoparticles and liposomes are primarily used to adsorb and absorb drugs of interest and even for encapsulating the sensitive therapeutics. Targeted drug delivery requires binding of biochemical molecules which offer directed control of therapeutic action. For continuous and responsive drug delivery system, thin films and even nanoparticles may be used as they can respond to the physiochemical changes that may occur in the body. Hydrogels form a three-dimensional structure consisting of cross-linked networks of water-soluble polymers, which can undergo conformational changes once they interact with water [104]. They can further be modified to react at a certain temperature, detection of analyte based on interaction with functional groups or pH in relation to their mode of action and target site. Upon reaching a certain site of action, the swelling dynamics will change, allowing for the diffusion of a therapeutic from the network matrix.

The fabrication of these systems relates to their chemical properties. If a system is designed for targeting the gastric intestinal tract, it must withstand physiochemical changes such as pH and temperature before it reaches its required site of action. Polymers such as chitosan, polyvinyl alcohol and ethylene glycol, can be used for both targeted and responsive action. Chitosan as a drug carrier has been used for various administration routes such as oral, bucal, nasal, transdermal, parenteral, vaginal, cervical, intrauterine and rectal [105]. As a responsive or targeted drug delivery vehicle, these biomaterials can be cross-linked or conjugated to other compounds to offer a responsive and improved targeting. Synthesis can be conducted by means of modifying temperature, ionic strength and pH during formulation. Physiochemical interactions such as hydrophobic/hydrophilic interactions, charge condensation, and hydrogen bonding have effects on the physiological interactions of the device.

Biodegradation relates to biocompatibility as the byproducts must be excreted or recycled by the body. Degradation and drug release kinetics are dependent on the concentration of the polymers and the cross linkers used. Cross linkers affects the drug release due to changes in porosity and viscosity. This can be changed by means of chemical modification, employing other compounds such as salts and metals. Salts can affect the tolerance of the physiochemical environment by changing the ionic strength of the device and act as cofactors for enzymatic action [129]. Different forms of drug delivery systems are designed based on mode of action. More focus has been directed towards responsive and target drug delivery towards organs. The brain and spinal cord are protected by the blood brain barrier [130]. This barrier affects the treatment of the neurological illness. By having different forms of drug delivery systems which may though have advantages and disadvantages, will offer a chance to design new therapeutic treatment methods.

Most molecules used in treatment of brain illnesses never make it pass through the blood brain barrier. This is due to the blood brain barrier that prevents the entrance of any form of exogenous substances to the brain and spinal cord [131]. The endothelial barrier which is linked to the brain astrocytes only permits the carrier mediated transport, active influx and receptor mediated transport via the BBB transporters [132]. This creates a problem for medical intervention when dealing with illnesses associated with the brain. For instance, it is only depression, schizophrenia and insomnia that have been found to have less problems in treatment when compared to illnesses such as Parkinson's disease (PD), brain cancer or stroke for which there is a limitation in terms of crossing the BBB due to the large size of drugs employed [133]. Advancements in improving drug delivery system would improve tribulations associated with BBB disruption e.g., local ultrasonic irradiation and usage of noxious agents which allows for the leakage of plasma proteins into the brain [134].

**Figure 11.**
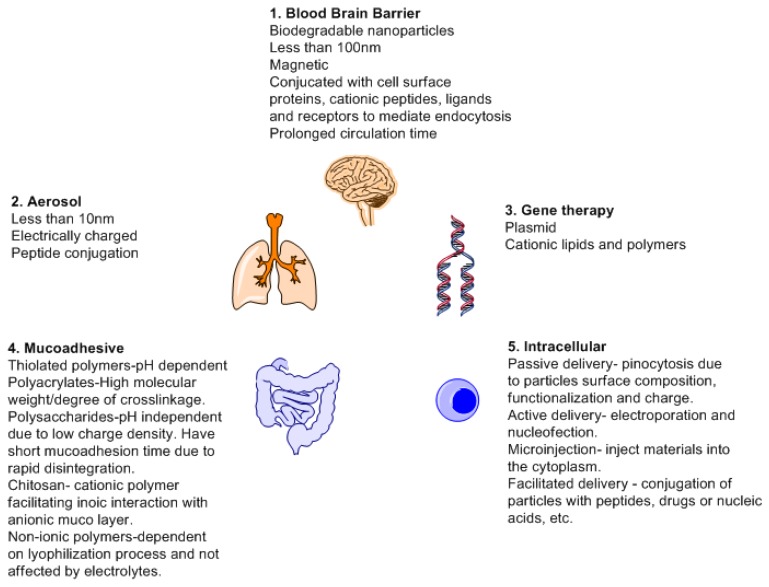
Nanoparticles as target specific drug delivery system. **1**. Blood brain barrier [137]; **2**. Aerosol [138]; **3**. Gene therapy [139]; **4**. Mucoadhesion [140]; **5**. Intracellular [141].

A drug delivery system has a number of benefits such as reduced toxicity, reduced side effects profile, controlled drug release, targeted drug delivery and usage of biocompatible (nonpathogenic such as viral vectors and additives for drug stabilization) substances. Nanoparticles are the most widely used since they can offer a number of benefits. The main benefits of nanoparticles being high surface to mass ration, quantum properties (conductivity), and ability to absorb and carry a variety of therapeutics [135]. Nanoparticles based on their mode of synthesis can have two major applications; imaging and carriers. As carrier, all forms of drug delivery must take into consideration and fulfill basic requisites such as knowledge of drug incorporation and release, formulation stability, shelf life, biocompatibility, biodistribution, targeting and functionality before they can be declared fit for medical use or FDA approved [136]. Figure 11 is a schematic depicting different properties required and targets of drug delivery system.

## 5. Integration of Biosensors with Drug Delivery Systems

Biosensors are the tools that can shape illness treatment by increasing accuracy of diagnosis, illness monitoring and prognosis. The advantages of biosensors are that they are easy to use, inexpensive, rapid, robust and can allow analysis of different biomarkers simultaneously [142]. The other main advantage is that there is no sample preparation since the biosensor can detect the biomarker within a pool of other bimolecular substances and this makes the integration of biosensors with current drug delivery systems feasible. Microneedles are painless minimally invasive drug delivery systems that do not contact with blood thereby reducing infection and risk of device contamination. In drug delivery, these microneedles are used to inject a therapeutic transdermally whilst for biomedical sensing they aid in fluid extraction for analysis. Utilizing such and many other tools the current research in illness management focuses one of its aspects on integration of biosensors with drug delivery systems. Many such systems that have been studied and published are based on responsive drug release, biocompatibility, biofouling, self-regulatory implants and refillable reservoirs [143,144].

### 5.1. Bio-Micro-Electro-Mechanical Systems (Bio-MEMS)

**Figure 12.**
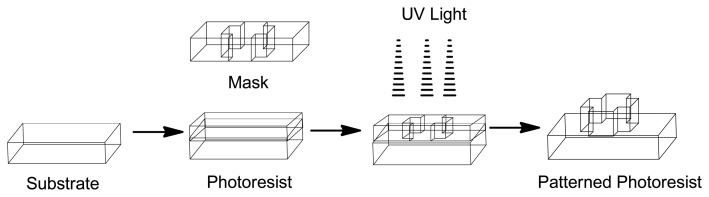
Photolithography process (Adapted from [147]).

The development of Micro-Electro-Mechanical Systems (MEMS) devices is accomplished the process of micro-fabrication, where silicon, glass and plastic are used. The initial stage for designing MEMS device is patterning technique where photolithographic process is used to design desired patterns on the wafer surface (Figure 12). The wafer is photoresist and then exposed to radiation through a mask which contains the pattern of interest. Once a pattern has been formed the photoresist is removed. The next step is deposition process were a thin film of material (bioeletrics, polymers (polydimethylsiloxane (PDMS) and polymethylmethacrylate (PMMA)), silicon dioxide, silicon nitride, metals (electrodes) or biomolecules) is deposited on the surface of the wafer [145]. This is followed by the process of etching which can be either wet where etching is due to liquid chemicals or dry where gas-phase chemistry is used. In both the phases etching processing can occur in all directions equally leading to mask undercutting and a rounded etch profile (isotropic) or be directional (anisotropic) due to either chemical or physical induction [146]. The final step is boding where the two substrates are bound together by anodic or fusion bonding [147]. The use of MEMS has led to the development of microfluidics which is a field of the design and development of miniature devices that can sense, pump, mix, monitor and control flow of small volumes of fluids [148]. Table 3 briefly summarizes the areas of BioMEMS application and examples of commercially available BioMEMS products and prototypes.

BioMEMS technology has allowed fabrication of both disposable (external application) and implantable drug delivery systems and diagnostic tools. Solid durable, solid degradable and hollow microneedles can be used for delivery of insulin (JewelPump, Debiotech) and for vaccination (Intaza, Sanofi Pasteur) [149]. Implantable drug delivery microdevices designed by means of BioMEMS technology can reduce conventional implantable drug delivery devices disadvantages. Most implantable drug delivery devices have unintended drug dumping events which cause side effects and reduce patient compliance as this causes health risk to patients [150]. Implant lifetime also affects compliance as this increases cost of implant replacement. These implants have further problems such that the implant drug release rate and drug contents cannot be changed without invasive procedure. Conventional pumps are usually osmotically, electrolytic or peristaltic driven [151]. By means of BioMEMS, a piezoelectric pump controlled drug delivery system was made for transdermal delivery of insulin by means of using microneedle, which improved precision and accuracy in relation to mechanical controlled pumps [152]. For longer lifetime and improved biocompatibility, the BioMEMS device will require use of biodegradable polymers or compounds that mitigate tissue response to the implant such as antibiotics or anti-inflammatory agents [153].

### 5.2. Smart Polymers

**Figure 13.**
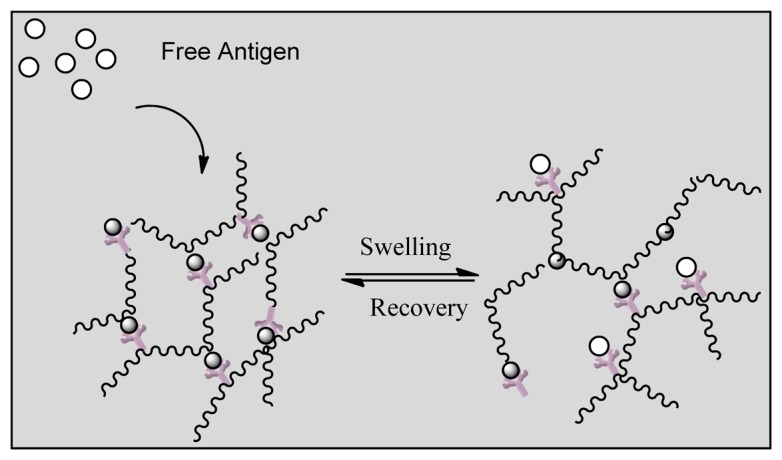
Reversible antigen responsive hydrogel (adapted from [114]).

Smart polymers represent a group of polymers that function in the same manner as biological systems. Stimuli responsive hydrogels can undergo structural changes when exposed to external stimuli such as pH, temperature and ionic changes. The polymers are divided into three groups based on their physical form. Linear free chains in solutions are when the polymer undergoes a reversible collapse after a stimulus is applied, covalently cross-linked reversible gels are when swelling/shrinking are triggered by environmental changes and chain adsorbed/surface-grafted form represent polymers that have reversible swelling/collapse on the surface once a trigger is changed [154,155]. Similar to affinity biosensors a hydrogel has been designed by grafting an antigen-antibody complex onto polymer network that will lead to competitive binding of the free antigen triggering a change in the network structure of the hydrogel [114]. Figure 13 indicates that the hydrogel regains its primary structure due to shape memory behavior after reversible binding [156]. Such behavior allows long term use of the system unlike affinity biosensors that get saturated over time as reversible binding is not favored. In another approach the entrapment of glucose oxidase within a pH responsive hydrogel (gluconic acid increase due to oxidation of glucose) and attachment of insulin allowed the smart polymers to act as both drug delivery vehicles for insulin in addition to being a biosensor of glucose concentration [157]. Other reversible systems include desthiobion/biotin and concanavalin A immobilized systems. Desthiobion/biotin-binding protein complex can be dissociated under physiological conditions by either biotin or desthiobiotin (analogue of biotin) [158]. Since biotin can be used to label a variety of proteins, this can be conjugated to either antibodies or antigens to serve as a reversible biosensor. Immobilization of Con A has shown to lead to a reversible sol-gel phase in the presence of free glucose again due to competitive binding with insulin conjugated to glucose [159].

### 5.3. Microfabricated Devices

Most of the microfabricated devices are in the form of biosensors. There is a time limitation to the use of microfabricated implantable biosensors due to their short time of functionality. Designing an implantable biosensor that has long term functionality can be a critical component of the ideal closed-loop drug delivery or monitoring system, without considering issue of implant biocompatibility and biofouling which must be addressed in order to achieve long-term *in vivo* sensing [160]. By use of a thermal, pH, ionic strength or biomolecular sensitive hydrogel as a transducer this can be implied in integration of drug delivery system and biosensor technology with better biocompatibility and reduced biofouling.

A cantilever can be employed as a lid on a reservoir whereby a sensing molecule embedded in a responsive hydrogel can stimulate the opening and closing of the lid in relation to analyte quantity. Furthermore the electrically responsive hydrogel can be used as components of MEMS-based sensors or drug delivery devices whereby the external electrical current can be applied on an implant to stimulate drug release intramuscularly. For drug delivery MEMS technology has been applied to formulate microparticles and micro-reservoirs.

**Figure 14.**
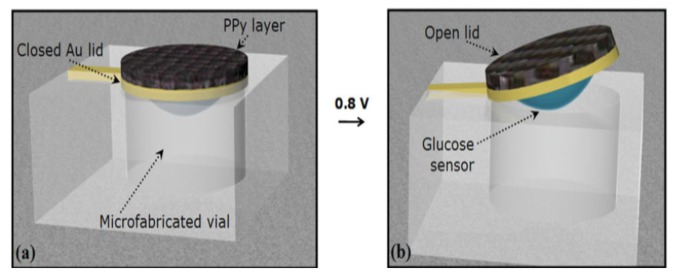
Micro-reservoir and microvalves in microfluidics technology (adapted from [162]).

Microparticles have been formed by means of generating a pattern of wells ranging in size from 25 to 100 μm inside silicon squares ranging from 80 to 150 μm in size [161]. These wells are then filled with a drug of interest and then sealed with a dissolvable cap that has bioadhesive properties for targeted delivery. These microparticles can be further improved by use of smart polymers that can shrink when an analyte is detected as caps to facilitate responsive drug release, thus integrating with biosensor. The micro-reservoirs in Figure 14 have been made out of silicon and covered with a gold membrane which is stimulated by a voltage to rupture the membrane [140]. Instead of voltage, smart polymers can be used to collapse in response to analyte concentration or by means of generating conductive polymers that can be stimulated during redox reactions. Microfabricated devices have led to the development of controlled release microchips.

### 5.4. Lab-on-A-Chip

**Figure 15.**
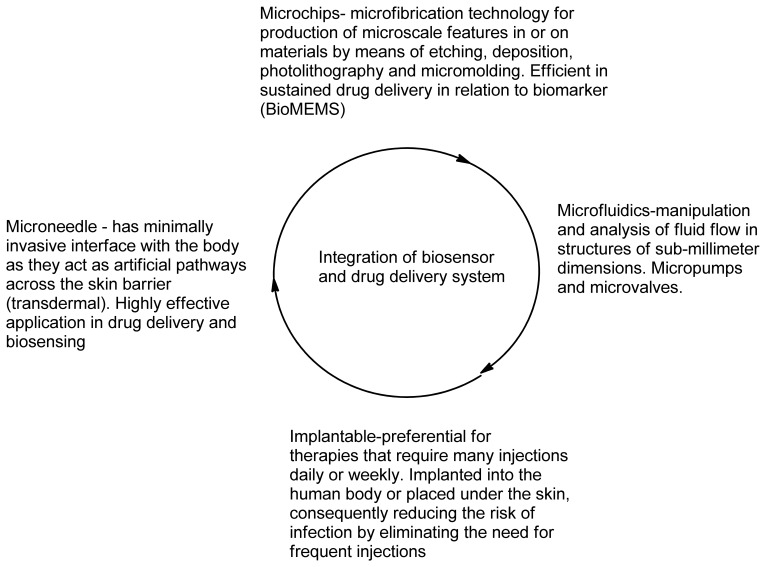
Technologies for integration of biosensors and drug delivery systems.

Lab-on-a-chip systems are increasing rapidly as they have significant benefits in different fields of health care and environmental affairs [163]. These benefits include rapid data analysis, improved analysis and portability of the devices. This allows individuals to monitor their own health sparing them from visiting a physician. Since technologies such as lap-on-a-chip can generate data comparable to a laboratory conducted data; this allows point of care diagnosis and treatment. Incorporation of a micro-reservoir drug depot, micro-pump, valves, and sensors onto BioMEMS devices allowed responsive and controlled release of drug. Controlled release is required as many drugs delivered through conventional modes of delivery leads to low bioavailability with low concentration and increase toxicity when high drug concentration is released or accumulates over time. A controlled-release microchip has been created that use silicon wafers and different drug depots for single and multiple drug release [164]. Integration of biosensors and drug delivery can be achieved by adding drug loaded hydrogels, biosensors, and other features that are responsive to the local environment that ultimately allows pharmaceutical devices to operate in a more closely integrated manner with the biological surroundings with limited scientist intervention (Figure 15).

Microchips are fabricated through the use of MEMS technology by first selecting a biocompatible substrate and etching of micro-reservoirs which will hold therapeutic solutions. Next is the selection of the conductive sealant (thin membrane) which serves as an anode as well. The choice of membrane is such that it does not dissolve/rupture in a solution in the absence of an applied electrical potential. For *in vivo* implantation one has to take into account the presence of oxygen and chloride ions which lead to corrosion of metals [165]. Microchips have advantages ranging the ability to pattern multiple micro-reservoirs which can hold multiple drugs; this prevents mechanical breakdown or leakage of drug during incomplete closure of the lids due to lack of moving parts such as glucose biosensor; eliminates patient or doctor intervention for functionality and can offer a close loop system when integrating biosensors ([166]. The design of disposable chips can also be beneficial in both biosensor and drug delivery system. Microneedles offer a non-invasive drug delivery and biosensing advantages compared to implantable systems. These can be used in combination with MEMS technology, utilizing micropumps that would allow continuous drug delivery [167]. This technology can be applied to vaccination and chronic pains whereby the responsive microneedle chip can be placed transdermally to release a certain level of therapeutics corresponding to the amount induced by analyte thereby increasing bioavailability and reducing localized toxicity as the therapeutic will release in relation to analyte concentration.

## 6. Conclusions

According to the World Health Organization, cardiovascular diseases are the leading cause around the World for an estimated 12 million deaths. Diabetes mellitus is however categorized on a pandemic level where its prevalence in Africa ranges between 1 and 20%. The increase in chronic respiratory diseases is often under diagnosed due to limited diagnostic resources. The cause in children is mainly due to allergens and pollutants which can be monitored and controlled. Due to low availability and accessibility of drugs and diagnostic tools, these diseases continue to increase. Integration of biosensors with drug delivery builds the design of implantable pharmacy which can operate as a closed loop system. This will offer continuous diagnosis, treatment and prognosis without vast data processing and specialist intervention. Point of care treatment moving from lab-on-a-chip technology to implantable chips which interacts with drug reservoirs, will increase compliance of patients who require continuous monitoring as in case of chronic diseases such as diabetes, lupus, osteoarthritis, rheumatoid arthritis, cancer, Cystic fibrosis, asthma and Parkinson's disease, coronary heart illness and AIDS. Implantable sensors are expected to interface with the body's biochemistry which will provide a critical link between diagnosis and therapeutics. Thus allowing continuous monitoring of analyte concentration and rapid analysis before major physiochemical outburst can occur such as hypertension. However, the creation of biosensor integrated drug delivery system requires a closed loop monitoring of the device. The use of implants in a BioMEMS category can provide a continuous drug supply at a specified time interval to allow better illness management without any denting intervention. Illnesses such as diabetes and coronary heart diseases, asthma, and arthritis require a responsive treatment since physiochemical changes may occur anytime.

In general, integration of biosensors and drug delivery systems offers patients a chance for self-monitoring which will improve illness management since all information in respect to their medical problems may be continuously monitored and maintained. Early detection of chronic illnesses such as cancer will therefore offer better and effective therapeutic treatments, while illness monitoring is applicable to common chronic illness such as diabetes and cardiovascular diseases which are increasing at an alarming rate in developing countries. By designing an implantable biosensor which will function as a “lab on a chip” will facilitate rapid illness management since the patients are in control of the health status. This may further be optimized by including multiple drugs in the implant reservoir for better illness management, thus preventing any further complication that may occur during self-regulatory therapeutic treatment

## Figures and Tables

**Table 1.** Groups of biosensors based on transduction signal, their mode of detection and applications.

**Transduction**	**Mode of Detection**	**Application**	**References**
Optical	*Surface Plasmon Resonance*-immobilizes antibodies/ligands/receptors. The analyte concentration is measured upon adsorption	Hand held refractometer (Rhino Series, Reichert, Inc., USA). Can detect analytes in urine	[57,58]
	*Fluorescence*- chemical, enzymatic and cellular changes by means of probing	Fluorescence Resonance Energy Transfer-Protein and nucleic acid analysis (Invitrogen, USA)	[59]
	*Ellipsometric*- reflection of a light beam from a reflective surface in relation to adsorbed analytes	Nanofilm_ep3seAccurion, USA. Spectrometric measurements. Binding of analyte to surface	[60]
Thermal	*Calorimetric*- measures a change in temperature in the solution containing specific analyte and converts it into concentration	Auto-iTC_200_ system (GE Healthcare, USA) and DSC used for characterizing molecular interactions/ enzyme kinetics	[61]
Mass sensitive	*Surface acoustic wave*- generate and detect acoustic waves using inter-digital transducers. This will detect changes on the surface, such as mass loading, viscosity and conductivity changes	VaporLab, Microsensor Systems, USA. Gas analysis on film swelling results in electrical signal. Breath analysis of volatiles	[62]
	*Quartz crystal microbalance*-consists of a thin quartz disk with electrodes plated on it. Measures a mass per unit area by measuring the change in frequency of a quartz crystal resonator	QCM200, Stanford Research Systems, Inc, USA; Attana Cell 200. Can measure specific analyte concentration	[63,64]
	*Cantilever-* nanomechanical biosensors microfabricated with the standard silicon technology. The surface is coated with detectors, which will cause the cantilever to bend once binding occurs	MEMS (i-STAT®, Abbott Laboratories, USA). Measures concentration and analytes in body fluids	[65]
Electrochemical	*Conductance*- conductive properties of medium between two electrodes (ionic strength changes)	Enzymatic reactions yielding charged substances. Enzyme field-effect transistor (EnFET); Nanowires	[66,67]
	*Amperometric/Voltammetry*-generated current during redox reaction	Electrochemical ELISA (Thermo Scientific, USA)	[68]
	*Potentiometric-* charge accumulation or potential (ionic)	FreeStyle Navigator Continuous Glucose Monitoring System (Abbott Laboratories, USA). Glucose/cholesterol levels; Ion-selective field-effect transistor (ISFET)	[67,69,70]
	*Impedance*- measure both resistance and reactance (change from weak or non-charge substances to highly charged)	Field-effect Transistor (FET). Drug effects on cell based ionic signatures (IQ Scientific Instruments, Inc); Single–walled carbon nanotubes (SWCNTs) Field-effect Transistor; Aptamer–modified carbon nanotube–FET	[71–73]

**Table 2.** Classification of drug delivery system platforms.

**Drug Delivery Systems**	**Application**	**Advantage/Disadvantage**	**References**
Nanosystems	Metallic nanoparticles - gold, silica, copper, silver	Magnetic resonance imaging and photothermal ablation of cancer cells. Carriers can cross the BBB.	[110,111]
	Polymeric nanoparticles - synthetic/natural polymers such as/lipid/proteins	Biodegradable, surface modification for targeted and responsive drug delivery and biocompatibility.	[112]
	Carbon nanotubes	High propensity to cross cell membrane via endocytosis. Can deliver therapeutics in a form of peptides and nucleic acids.	[113]
Hydrogels	Water soluble polymers-cross linked	Highly porous, biodegradable and deformable. Low tensile strength leads to premature dissolution.	[108]
Stimuli responsive polymers	pH responsive	pH fluctuation	[114,115]
	Temperature responsive	Temperature fluctuation	[111,115]
	Electroconductive polyaniline, polyacetylene, polypyrrole, polythiophene and their derivatives	Act as transducers for the concentration of analyte to be conveyed electronically. Biocompatibility is questionable.	[117,120, 121]
	Biochemical-antigen or analyte responsive (reversible binding using antibodies/receptors; molecularly imprinted)	Swelling of reversible molecular imprint due to antigen/analyte binding. Management of biochemical imbalances/detection of foreign particles. The molecular imprint reduces cost of using macromolecules for sensing analyte.	[116,117]
Liposomes	Self-assembling spheres composed of lipid bilayers	Biocompatible and can deliver sensitive therapeutics (DNA). Engulfed by endoreticulum.	[99]
Viral and bacterial vectors	Adenovirus, retrovirus and adeno-associated virus	Manipulation of viral mode of nucleic acid delivery into host nucleus, cell specific infection and gene expression. Recombination event results in modification of the viral vector into a pathogen.	[118]
	Bacterial ghost- empty bacterial envelopes of Gram-negative bacteria	These offer natural target specificity function as they constitute all bio-adhesive surface properties.	[119]
Micelles	Polymeric micelles - Amphiphilic copolymers	Suitable for water-insoluble drug. Useful for targeted drug delivery.	[120]
Dendrimer	Scaffold - multiple highly branched monomers emerge from central core	Modifiable surface allowing easy conjugation for target specificity and multiple drug conjugation.	[121]
Cells	Transduced cell- stem cells, progenitor cells and fibroblast	Transduced cell allow gene expression in individuals with genetic disorders. Disadvantage is gene integration which interrupts gene expression of other genes.	[122]
	Cell carriers-macrophages and red blood cells	Macrophages can bind to cells, macromolecules/foreign particles. Red blood cell used for transporting antiviral/antimicrobial/anti-inflammatories.	[123,124]
Soluble macromolecules	Loligomers and peptide-based matrices (cell penetrating peptides) -synthetic and recombinant	These are used as most peptides have been shown to traverse biological membranes.	[125,126]
Drug Nanoparticles	Drug nanocrystals- Rapamune®, Emend®, TriCor 145®	Poorly soluble drugs, carriers with biocompatibility and biodegradation problems are not required.	[127,128]

**Table 3.** Applications of BioMEMS.

**Applications**	**Devices and Manufacturers**
Detection	Rapi*D*x (Sandia National Laboratories)
Analysis	Lab on a Chip (STMicroelectronics)
Diagnosis	Piccolo^®^ Xpress (Abaxis)
	GeneChip^®^ **Microarrays** (Affymetrix)
Therapeutics	Argus™ Retinal Prosthesis System (Second Sight)
Drug delivery	MiniMedParadigm^®^522 insulin pump (Medtronic Diabetes)
Microneedles	Nanopatch™ (Vaxxas)
